# Metabolic effects of an AT1-receptor blockade combined with HCTZ in cardiac risk patients: a non interventional study in primary care

**DOI:** 10.1186/1471-2261-8-30

**Published:** 2008-11-10

**Authors:** Peter Bramlage, Eleonore Schönrock, Peter Odoj

**Affiliations:** 1 Institute for Clinical Pharmacology, Medical Faculty Carl Gustav Carus, TU Dresden, Germany; 2 General Practitioner, Potsdam, Germany; 3 General Practitioner, Kirchlengern, Germany

## Abstract

**Background:**

The reduction of blood pressure alone does not eliminate the increased risk of arterial hypertension. Whilst concomitant metabolic risk factors have been shown to be responsible, the available pharmacotherapy has differential effects on these metabolic risk factors. For example, diuretics and betablockers worsen glucose metabolism, hence the starting point of the current subanalysis of the CHILI (Candesartan in patients with HIgher cardiovascuLar rIsk) study was the assumption that an angiotensin receptor blocker may counterbalance the metabolic effects of a low dose diuretic in patients with several metabolic risk factors.

**Methods:**

The present study was performed as a non-interventional observational study in Germany. Patients with previously uncontrolled hypertension with at least one further risk factor in which physicians deemed a treatment with 16 mg Candesartan/12.5 mg HCTZ to be necessary were included. The risk factors were calculated in patient subgroups with diabetes, the metabolic syndrome (MetSyn) and neither condition (control). The risk of cardiovascular mortality within the next 10 years was calculated using the SCORE algorithm of the ESC.

**Results:**

Between August 2006 and February 2007 a total of 3,787 patients were included into the non-interventional trial. Patients were 62.2 ± 11.3 years old, 48.1% were female, 97.5% had at least one additional risk factor. Blood pressure was reduced by -27.2/-13.4 mmHg with only minor non significant variations between patient groups. Waist circumference was reduced (P < 0.0001) and HDL-C elevated (P < 0.05) in every subgroup except the control subgroup. Fasting blood glucose was reduced by -5.6 ± 21.6% (P < 0.0001 vs. baseline and vs. control) as well as triglycerides (-4.9 ± 29.4%; P < 0.0001 vs. baseline and vs. control). The SCORE value was reduced substantially (-8.7, -3.2 and -2.7% in patients with diabetes, the metabolic syndrome or neither).

**Conclusion:**

The present study demonstrates that a 16 mg candesartan/12.5 mg HCTZ based treatment results in a pronounced blood pressure reduction and was associated with a favourable change in metabolic risk factors such as HDL cholesterol, triglycerides and blood glucose. These data indicate that metabolic effects observed in clinical trials like ALPINE, SCOPE or CHARM can also be observed in an unselected patient population in primary care.

## Background

Arterial hypertension is one of the most prevalent risk factors for cardiovascular disease [1-3] and an important cause of death worldwide [4]. The relationship between blood pressure and cardiovascular risk is almost linear in patients without end organ damage [5,6] and an arbitrary threshold of 140/90 mmHg has been defined to simplify diagnosis and treatment approaches in daily practice [7]. Lifestyle interventions have been proposed to lower blood pressure and cardiovascular risk [8], but they often fail to be long-term solutions. Therefore a number of anti-hypertensive drugs have been developed, giving clinicians many options. Whilst lowering BP is a mainstay of cardiovascular risk reduction, it is important to treat the other cardiovascular risk factors as well [9].

Hypertension is frequently found together with other metabolic risk factors (low HDL-Cholesterol, abdominal obesity, high triglycerides, high fasting blood glucose etc.). The HOT study for example found that each of the risk factors considered was an important cause of residual risk, despite good blood pressure control [9]. It therefore seems to be of principal importance to address other correctable risk factors and to be particularly conscious about the metabolic effects of antihypertensive drugs even in patients being treated to goal (120/80 mmHg).

In a recent meta-analysis Elliott and colleagues [10] showed that increased blood sugar values and the subsequent development of diabetes occur more often in patients receiving diuretics and betablockers instead of angiotensin receptor blockers (ARBs) and angiotensin converting enzyme (ACE) inhibitors. Furthermore it was recently demonstrated that patients on betablockade have more difficulties to reduce abdominal obesity and additional metabolic risk factors than those patients on a combination of renin-angiotensin system (RAS) blocking agents and Calcium Channel Blockers (CCBs) [11]. Of note, the ARB, candesartan reduced the number of patients developing diabetes in the CHARM [12], SCOPE [13] and ALPINE: [14] trial. When candesartan was administered to a group of hypertensive subjects it caused a reduction in C-reactive protein and an increase in adiponectin and markers of insulin sensitivity; as measured by QUICKI (Quantitative Insulin-Sensitivity Check Index) index [15].

Of particular interest within this context is a combination of candesartan with a low dose diuretic (12.5 mg hydrochlorothiazide, HCTZ) and to test whether the properties of both agents combine to create a beneficial outcome for patients. It was therefore the aim of the present analysis of the non interventional CHILI (Candesartan in patients with HIgher cardiovascuLar rIsk) study to investigate the influence of candesartan in fixed combination with HCTZ on a number of metabolic parameters and cardiovascular risk factors in more detail. The study was conducted in a primary care setting in order to acquire the broadest possible spectrum of patients in clinical practice. Three core questions were to be answered: 1) reduction in blood pressure; 2) reduction of cardiovascular risk factors like fasting glucose, waist circumference, triglycerides, HDL-Cholesterol after 8 week treatment and differential effects in patients with diabetes, the metabolic syndrome and neither disease state; 3) change in the absolute 10 year risk of cardiovascular morbidity (using the SCORE method) and the contribution of a) blood pressure and b) metabolic effects to this effect.

## Methods

The present non-interventional study was conducted between August 2006 and February 2007 with the help of 893 primary care physicians all over Germany. Therefore a sample of general practitioners, internists, cardiologists and practical physicians was drawn from all segments of the Institute for Medical Statistics (IMS, Frankfurt, Germany). The study was duly notified according to local laws and regulations (§ 67 (6) Arzneimittelgesetz, AMG) to the higher authorities (Bundesinstitut für Arzneimittel und Medizinprodukte, BfArM) and the federal panel doctors' association (Kassenärztliche Bundesvereinigung, KBV). Approval was obtained by the Technical University Dresden ethical committee. Due to regulations for this kind of study no patient informed consent had to be obtained. Methods of this study have been published previously [16].

### Patients

Patients were at least 18 years old, had essential arterial hypertension with blood pressure values beyond 140/90 mmHg (> 130/85 mmHg in diabetic patients) and previous antihypertensive therapy of at least 8 weeks duration had proven unsuccessful. In addition to this one of the following risk factors had to be present: Diabetes mellitus, dyslipidemia, abdominal obesity, hs-CRP ≥ 2 mg/l or confirmation of microalbuminaria (MAU). Patients who fulfilled these criteria and who's treating physician deemed a treatment with 16 mg Candesartan/12.5 mg HCTZ to be necessary were documented.

### Metabolic Syndrome definition

The AHA/NHLBI 2004 definition was used to determine the presence of the metabolic syndrome [17]. It requests that 3 out of the following 5 criteria are met: 1) waist circumference > 102 cm in men and > 88 cm in women; 2) blood pressure readings ≥ 130 mmHg systolic or ≥ 85 mmHg diastolic; 3) fasting glucose ≥ 5.6 mmol/L (≥ 100 mg/dL) or known diabetes mellitus; 4) triglycerides ≥ 1.7 mmol/l (≥ 150 mg/dL); 5) HDL-Cholesterol < 1.0 mmol/L (40 mg/dL) in men, and < 1.3 mmol/L (50 mg/dL) in women.

### Calculation of the SCORE value

The following parameters were used for the calculation of the score [18]: sex, age, HDL-C, total cholesterol (either indicated or calculated by the Friedewald formula), systolic BP and smoking status. In the presence of diabetes, the score was increased by a factor of 2 in men and by a factor of 4 in women. Calculations were done without replacement of missing values. To calculate the blood pressure risk reduction attributable to diabetes, the baseline SCORE risk was modified by replacing the blood pressure baseline value by the 8 week follow-up value but keeping the baseline values for metabolic risk factors. To calculate the risk attributable to metabolic changes, baseline SCORE risk was modified by replacing the baseline values of metabolic parameters by the 8 week values but keeping the baseline blood pressure within the model.

### Study conduct

Physicians were asked to document at least 3 patients with arterial hypertension and concomitant cardiovascular risk factors. The observational period was 8 weeks and patients had a voluntary additional visit at 4 weeks and the case report forms were retrieved by the CRO Christine Franzen Consulting, (Stolberg, Germany) and screened routinely for plausibility and completeness. Source data verification was however not performed. Confidentiality: Patient data were recorded anonymously (age and gender only). Electronic data processing was conducted in accordance with local laws and regulations and the participating GPs received remuneration for the documentation of each patient which was in accordance with the "Gebührenordnung für Ärzte" (GOÄ).

### Statistical Analyses

Regarding safety, the trial was adequately sized (n = 3,787) to identify rare AEs, i.e. those that may not have been detected in previous clinical studies, (incidence 1: 1,000) with a probability of > 99%. These data have been published previously [16]. The statistical analysis was performed descriptively and was interpreted in an explorative way. Comparisons were made for a number of variables and analyzed using descriptive statistics. The number of patients is given for each value and differences were calculated in patients with values at baseline and follow-up (per protocol). The analysis of data was performed with ACCESS 2003 and Winstat for Microsoft Excel. Tests applied are indicated in the legends of tables and figures.

## Results

3,787 patients were included into the present study. The patients had a mean age of 62.2 ± 11.3 years, 48.1% were female and BMI was 29.5 ± 4.7 kg/m^2^. 97.5% had at least one cardiovascular risk factor, 29.8% existing cardiovascular disease. 13.0% of patients had angina pectoris, 12.5% heart failure, 8.4% myocardial infarction, 6.2% kidney failure and 4.9% previous stroke.

### Cardiovascular risk factors over time

The risk factors making up the metabolic syndrome were elevated throughout all patient subgroups (metabolic syndrome, with diabetes mellitus and neither condition) with hypertension (inclusion criterion), dyslipidemia (78.2%) and abdominal obesity (65.4%) being the most frequent. Tables 1, 2 and 3 display the course of these risk factors throughout the study in patients with the metabolic syndrome, with diabetes mellitus and neither condition. Blood pressure was significantly reduced in all patients (-27.2/-13.4 p < 0.0001) and there was no significant difference between the extent of blood pressure lowering (p = n.s.) between groups. Fasting blood glucose and triglycerides were unchanged in patients without the metabolic syndrome or diabetes only (p = n.s.); every other parameter was significantly changed versus baseline in a positive direction.

**Table 1:** Cardiovascular risk factors at baseline and follow-up in patients without the metabolic syndrome1 and diabetes mellitus^2^

		**Baseline**			**8 weeks**			**difference^2^**		
		**N**	**mean**	**± SD**	**N**	**mean**	**± SD**	**N**	**mean**	**± SD**
**Blood pressure**										
systolic	mmHg	354	159.8	14.7	348	133.0	10.8	341	-27.0*	14.5
diastolic	mmHg	353	93.3	9.3	348	80.7	6.0	340	-12.8*	10.1

**Fasting plasma glucose**										
	mg/dL	354	85.9	11.5	205	86.8	14.5	203	1.7	15.0

**Waist circumference**										
men	cm	141	97.9	10.4	135	96.8	9.7	133	-0.8*	1.9
women	cm	142	88.8	11.7	130	87.8	10.5	130	-0.6*	2.8

**Triglycerides**										
	mg/dL	351	138.0	80.6	197	137.7	57.6	195	0.3	3.86

**HDL Cholesterol**										
men	mg/dL	169	54.1	13.9	94	53.8	13.2	94	1.6	10.7

women	mg/dL	183	67.4	17.3	96	66.6	17.1	95	-0.7	15.8

^1 ^defined by the AHA/NHLBI 2004 [17]; ^2 ^diagnosis assigned by physician; ^3 ^difference versus baseline; statistical test applied: t-test; * p < 0.0001. Adjusted for age.

**Table 2:** Cardiovascular risk factors at baseline and follow-up in patients with the metabolic syndrome^1^

		**Baseline**			**8 weeks**			**Difference^2^**		
		**N**	**mean**	**± SD**	**N**	**mean**	**± SD**	**N**	**mean**	**± SD**
**Blood pressure**										
systolic	mmHg	1319	160.0	13.8	1312	133.3	10.4	1289	-26.6*	14.3
diastolic	mmHg	1319	94.6	8.6	1312	80.9	6.5	1289	-13.6*	9.2

**Fasting plasma glucose**										
	mg/dL	1234	95.5	17.9	769	92.9	14.9	764	-2.5*	13.1

**Waist circumference**										
men	cm	634	107.2	11.7	599	106.1	11.8	598	-1.1*	4.1
women	cm	530	97.3	13.0	498	96.7	13.3	496	-0.7*	3.5

**Triglycerides**										
	mg/dL	1186	205.0	91.3	753	185.0	74.4	741	-19.1*	61.0

**HDL Cholesterol**										
men	mg/dL	635	46.9	17.4	395	47.6	13.3	384	1.1*	11.0

women	mg/dL	526	51.3	16.6	338	53.2	15.6	331	2.3*	10.8

^1 ^defined by the AHA/NHLBI 2004 [17]; ^2 ^difference versus baseline; statistical test applied: t-test; * p < 0.0001. Adjusted for age.

**Table 3:** Cardiovascular risk factors at baseline and follow-up in patients with Diabetes Mellitus^1^

		**Baseline**			**8 weeks**			**Difference^2^**		
		**N**	**mean**	**± SD**	**N**	**mean**	**± SD**	**N**	**mean**	**± SD**
**Blood pressure**										
systolic	mmHg	1517	160.0	13.99	1489	134.1	11.2	1474	-25.9*	13.7
diastolic	mmHg	1517	93.4	8.46	1489	81.2	6.7	1474	-12.2*	9.0

**Fasting plasma glucose**										
	mg/dL	1302	132.0	36.8	994	121.8	31.4	972	-10.8*	30.1

**Waist circumference**										
men	cm	633	109.4	12.8	601	108.2	12.2	597	-1.3*	2.5
women	cm	570	100.6	14.7	530	99.5	13.6	526	-0.9*	2.7

**Triglycerides**										
	mg/dL	1187	205.1	104.5	834	185.6	78.4	811	-23.3*	76.2

**HDL Cholesterol**										
men	mg/dL	601	49.2	21.6	430	48.9	16.7	420	0.5**	14.36

women	mg/dL	558	54.0	19.0	373	54.0	17.7	361	2.0**	14.79

^1 ^diagnosis assigned by physician; ^2 ^difference versus baseline; statistical test applied: t-test; * p < 0.0001; ** p < 0.05. Adjusted for age.

### Comparison between different patient groups

**Figure 1:**
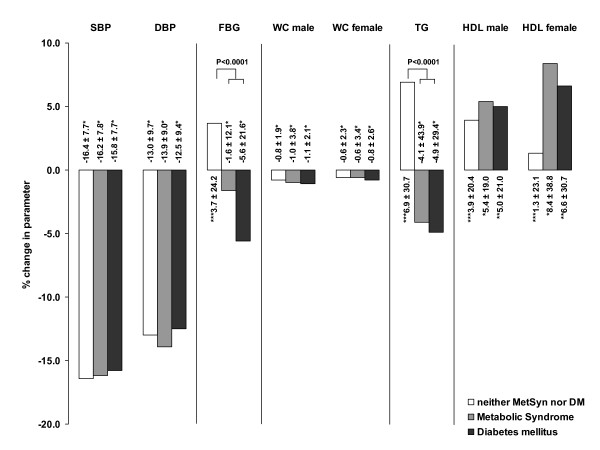
**Relative change in the single risk factors and of the metabolic syndrome in total**. The Metabolic syndrome is given, if 3 out of 5 criteria are met [17]. Significance: * P < 0.0001; ** P < 0.05; *** P = n.s.; Comparison between groups is a results of ANCOVA analysis (adjusted for age and gender); Differences vs. baseline are compared with a paired t-test (intraindividual comparison).

Figure 1 displays the relative change in risk factors in comparison between the three groups of patients. While fasting plasma glucose was unchanged (p = n.s.) in patients with neither the metabolic syndrome nor diabetes it was gradually reduced in patients with the metabolic syndrome (-1.6 ± 12.1%; p < 0.0001 vs. baseline) and strongly reduced in patients with diabetes (-5.6 ± 21.6%; p < 0.0001 vs. baseline). This corresponded to a mean absolute reduction of -2.5 ± 13.1 and -10.8 ± 30.1 mg/dL respectively. Triglycerides were similarly unchanged in the control group while they were reduced in MetSyn (-4.1 ± 43.9%; p < 0.0001 vs. baseline) and Diabetes (-4.9 ± 29.4%; p < 0.0001). Mean absolute values were -19.1 ± 61.0 and -23.3 ± 76.2 mg/dl respectively.

### Absolute cardiovascular risk in comparison to normal age matched controls

Finally absolute cardiovascular risk was calculated according to the SCORE method. The baseline risk differed substantially in the three patient subgroups (Diabetes 19.8 > MetSyn 7.4 > Neither 6.4%) as did the absolute risk reduction (Diabetes -8.7 > MetSyn -3.2 > Neither -2.7%). For details see table 4. The SCORE value was also calculated assuming that a) blood pressure was unchanged (risk reduction of -2.6, -2.9 and -7.6% in patients with neither, MetSyn or diabetes respectively) and b) that all other risk factors were unchanged (-0.4, -0.7 and -1.4) to estimate the proportion of risk reduction from the antihypertensive effect of candesartan (Table 4).

**Table 4:** SCORE risk at baseline and follow-up

	**Patients without diabetes^1 ^or MetSyn^2^**			**Patients with MetSyn^2^**			**Patients with diabetes^1^**		
	**N**	**mean**	**± SD**	**N**	**mean**	**± SD**	**N**	**mean**	**± SD**
**Total**									
**baseline**	225	6.4	6.0	885	7.4	6.7	818	19.8	14.4
**8 weeks**	139	3.5	3.1	576	4.0	3.5	607	11.2	8.2
**Difference^3^**	138	-2.7*	3.8	568	-3.2*	3.6	590	-8.7*	8.7

**Blood pressure alone**									
**baseline**	225	6.4	6.0	885	7.4	6.7	818	19.8	14.4
**8 weeks**	132	6.1	5.6	557	6.8	5.9	589	19.1	13.2
**Difference^3^**	132	-0.4*	1.2	555	-0.7*	1.4	578	-1.4*	3.3

**Risk factors alone**									
**baseline**	225	6.4	6.0	885	7.4	6.7	818	19.8	14.4
**8 weeks**	221	3.9	3.4	873	4.5	4.0	789	12.4	8.8

**Difference^3^**	219	-2.6*	3.4	862	-2.9*	3.3	786	-7.6*	7.7

^1 ^diagnosis assigned by physician; ^2 ^defined by the AHA/NHLBI 2004 [17]; ^3 ^difference versus baseline; * p < 0.0001.

## Discussion

Antihypertensive drugs have been shown to have differential effects on metabolic parameters in clinical trials and recent meta analyses [10]. Treatment with ARBs was associated with a reduced incidence of diabetes mellitus compared to placebo, diuretics and betablockers [10,19]. Consistent with this notion Law and colleagues recommend a combination of a standard dose of a RAS blocking agent with low doses of non-RAS blocking agents, as only the RAS blocking agents had no evidence of dose related adverse events [20]. The intention was to achieve substantial reductions in blood pressure with a reduction of drug related side effects. This approach has been well translated into the development of fixed dose drug-drug combinations. In the present non interventional study conducted in primary care a fixed dose combination of 16 mg candesartan with 12.5 mg HCTZ resulted not only in a strong reduction in blood pressure but also in a favourable change in metabolic risk factors. These changes were more pronounced in high cardiac risk patients like those with metabolic syndrome or Diabetes mellitus compared to patients with neither condition.

### Blood pressure reduction

In the present study a blood pressure reduction of -27.2/-13.4 mmHg was documented. Baseline blood pressure values were comparable throughout the subgroups as was the reduction in blood pressure induced by 16 mg candesartan/12.5 mg HCT. In a previous study patients with previously untreated severe hypertension (mean blood pressure 178/117 mmHg) who received 16 mg candesartan/12.5 mg HCTZ experienced a rapid blood pressure reduction that peaked at -38/-29 mmHg [21]. The blood pressure reduction in the present study matches well with previous data and extends these by the finding that patients previously uncontrolled will have a substantial reduction in blood pressure when prescribed the fixed dose combination. The absolute amount of blood pressure lowering (in mmHg) appeared to be dependent on baseline blood pressure but did not differ between patient types (diabetes, metabolic syndrome and neither condition). Therefore patients with a higher blood pressure value can expect a higher blood pressure lowering effect of candesartan/HCTZ.

### Reduction of cardiovascular risk factors

Throughout the study a reduction of cardiovascular risk factors was observed. However one should consider the short time frame (observational period 8 weeks) in which the changes were observed and its relative magnitude (Figure 1). While a slight reduction of waist circumference could be observed in all three patient groups, changes were non-significant with respect to fasting blood glucose, triglycerides and HDL cholesterol in patients that neither had the metabolic syndrome nor diabetes (eumetabolic). Patients with metabolic risk factors however showed a pronounced response in these metabolic risk factors when treated with candesartan/HCTZ. Previous studies including the ALPINE (OR 0.12; 95%CI 0.01–0.97 vs. Thiazides) and the combined SCOPE and CHARM studies (OR 0.80; 95%CI 0.67–0.95 vs. placebo) have already demonstrated the reduced diabetes incidence in patients taking candesartan [12,14,22]. In the randomized double blind ALPINE study newly diagnosed hypertensive patients on a low dose diuretic alone or in combination with a betablocker were compared in their use of candesartan without a calcium channel blocker and followed for a year [14]. At a similar level of blood pressure reduction, patients on the candesartan fixed dose combination had lower levels of plasma glucose, triglycerides and higher HDL-cholesterol. At 12 months, 18 patients in the hydrochlorothiazide group versus five in the candesartan group had a 'metabolic syndrome', as defined by the World Health Organization (P 0.007) despite 1 year of active blood pressure-lowering therapy.

The underlying mechanism for the eumetabolic effects of Candesartan are largely speculative. While there currently is great attention to the PPARγ activating properties of some ARBs [23] candesartan is less lipophilic [24] and probably does not directly activate PPARγ. It has however recently been reported that candesartan enhanced the gene expression of PPARγ and induced the increased expression of adipokines and a decrease in the pro-inflammatory cytokine TNF-alpha [25]. On the leptin contrary was reduced because of the repression of the Leptin gene by activated PPARγ. Whether this is an adequate explanation for the changes observed can only be speculated at present.

Previous studies in primary care, similar in design as the present study, have shown comparable results to the ones reported here [26,27]. These studies were substantially longer (6 and 9 month observations respectively) and point towards the conclusion that the observed effects of candesartan/HCTZ may well be even more pronounced longer term. Differential effects of ARB treatment between patients with and without the metabolic syndrome have also been observed in the study by Kintscher et al. [26], confirming the present result that severely metabolic patients (diabetes > metabolic syndrome) will particularly benefit from an ARB based combination treatment.

### Risk for cardiovascular mortality (SCORE)

Cardiovascular risk scores have been developed to estimate the cardiovascular risk of a given patient. They reflect findings from epidemiological studies like Framingham and PROCAM that a particular set of risk factors makes up the largest proportion of risk. The SCORE method has been developed under the auspices of the European Society of Cardiology (ESC) and has been adapted to fit particular countries in Europe [18] and specific risk tables for Germany have been published [28]. 5% has been suggested to be the intervention threshold for primary prevention by a working group of the German Society of Cardiology [29].

The documented baseline risk of in the present was well beyond the intervention threshold of 5% disregarding that most of these patients were eligible for intervention based on a diagnosis of diabetes mellitus or concomitant cardiovascular disease. Treating patients with the fixed dose combination of 16 mg candesartan/12.5 mg HCTZ resulted in a substantial risk reduction. Similar analyses have been conducted in the primary care setting in a previous study. Bramlage et al. showed that Irbesartan treatment (with or without HCTZ) led to a reduction of cardiovascular risk in hypertensive type-2 diabetic patients [30]. The effect was higher in men than in women but no specific data were reported for different patient subgroups. Also no effort was made to attribute SCORE reductions to different risk factors making up the score. Data from the present study point at an only partial responsibility of the blood pressure lowering effect (-27.2/-13.4 mmHg) but to the favourable metabolic changes induced by candesartan. This was unexpected given the dominant blood pressure lowering effect of the drug and it is also welcome based on earlier data from the Hypertension Optimal Treatment Study, that despite good blood pressure control, the residing metabolic risk factors were found to be an important cause of residual risk [9]. Whether or not a candesartan based treatment may narrow the gap between the cardiovascular risk of naturally normal and well controlled patients is however beyond the scope of the present study.

### Limitations

The present results have to be considered against the background of potential limitations. The study was not controlled and therefore the role of a placebo effect or the withdrawal of antihypertensive agents is unknown. Second, in the absence of a randomization procedure and not consecutive inclusion the influence of unknown biases, e.g. through patient selection, cannot be ruled out. Third, changes in concomitant medication influencing the metabolic profile (lipid lowering agents, oral antidiabetic agents or insulin) have not been documented in the present study. Fourth, randomly high values for blood pressure readings or metabolic changes may have resulted in artificially large reductions consistent with the phenomenon "regression to the mean", the extent of which is difficult to determine. Among the strengths of the study was the choice of the setting. Observational studies in primary care, which include typical patient groups and reflect current treatment approaches, are useful for complementing the findings of randomized controlled trials [31].

## Conclusion

The present study confirms prior results from randomized controlled trials that a candesartan 16 mg/HCTZ 12.5 mg based treatment resulted in a good population blood pressure control in actual practice. It further shows that despite concerns about the metabolic effects of diuretics, improvements in metabolic parameters may be seen in patients who receive a low-dose diuretic with candesartan as part of their overall medical care.

## Competing interests

Takeda Pharma GmbH, Aachen, Germany has financed the study. PB has received research support from Takeda for the statistical exploration, analysis and the preparation of the manuscript. PO and ES declare that they have no competing interests.

## Authors' contributions

PB has explored the data, requested statistical analyses from the CRO and wrote the manuscript. PO and ES have revised the manuscript for important intellectual content and have participated in the study conduct. All authors read and approved the final manuscript.

## Pre-publication history

The pre-publication history for this paper can be accessed here:


